# Brief psychological therapies for anxiety and depression in primary care: meta-analysis and meta-regression

**DOI:** 10.1186/1741-7015-8-38

**Published:** 2010-06-25

**Authors:** John Cape, Craig Whittington, Marta Buszewicz, Paul Wallace, Lisa Underwood

**Affiliations:** 1 Camden and Islington NHS Foundation Trust, St Pancras Hospital, 4 St Pancras Way, London NW1 0PE, UK; 2 Research Department of Clinical, Educational and Health Psychology, University College London, Gower Street, London WC1E 6BT, UK; 3 Research Department of Primary Care and Population Health, University College London Medical School, 2nd floor, Holborn Union Building, Archway Campus, Highgate Hill, London, N19 5LW, UK; 4 Health Service and Population Research Department, Institute of Psychiatry, Kings College London, De Crespigny Park, London SE5 8AF, UK

## Abstract

**Background:**

Psychological therapies provided in primary care are usually briefer than in secondary care. There has been no recent comprehensive review comparing their effectiveness for common mental health problems. We aimed to compare the effectiveness of different types of brief psychological therapy administered within primary care across and between anxiety, depressive and mixed disorders.

**Methods:**

Meta-analysis and meta-regression of randomized controlled trials of brief psychological therapies of adult patients with anxiety, depression or mixed common mental health problems treated in primary care compared to primary care treatment as usual.

**Results:**

Thirty-four studies, involving 3962 patients, were included. Most were of brief cognitive behaviour therapy (CBT; *n *= 13), counselling (*n *= 8) or problem solving therapy (PST; *n *= 12). There was differential effectiveness between studies of CBT, with studies of CBT for anxiety disorders having a pooled effect size [*d *-1.06, 95% confidence interval (CI) -1.31 to -0.80] greater than that of studies of CBT for depression (*d *-0.33, 95% CI -0.60 to -0.06) or studies of CBT for mixed anxiety and depression (*d *-0.26, 95% CI -0.44 to -0.08). Counselling for depression and mixed anxiety and depression (*d *-0.32, 95% CI -0.52 to -0.11) and problem solving therapy (PST) for depression and mixed anxiety and depression (*d *-0.21, 95% CI -0.37 to -0.05) were also effective. Controlling for diagnosis, meta-regression found no difference between CBT, counselling and PST.

**Conclusions:**

Brief CBT, counselling and PST are all effective treatments in primary care, but effect sizes are low compared to longer length treatments. The exception is brief CBT for anxiety, which has comparable effect sizes.

## Background

Anxiety and depressive disorders are common, with estimated combined prevalence varying between countries but over 10% in most Western countries [1-4]. The majority of such patients are treated in primary care, with few patients referred on to secondary mental health services [5,6].

With access to psychological therapies being limited [7], psychological therapy provided within primary care settings for depression and anxiety is usually brief [8]. In the UK, six sessions is a common treatment length [9]. This contrasts to the treatment lengths of 12 - 24 sessions which have been the subject of most efficacy trials of psychological therapies in secondary care settings [10,11].

Most reviews of psychological therapies combine primary and secondary care studies [11-13]. Recent reviews which have included analyses limited to primary care, have focussed on studies of patients with a diagnosis of depression [14-18] or studies of a specific type of psychological therapy [19]. Although studies of psychological therapies in primary care frequently include patients with both anxiety and depression, reflecting the heterogeneous patient presentations in primary care where mixed anxiety and depression is the most common diagnosis [20-22], such studies are excluded when the selection criteria for reviews are limited to single mental health diagnoses. The only reviews covering the range of mental health disorders and types of psychological therapies used within primary care date back over a decade [23-26].

This systematic review, meta-analysis and meta-regression includes studies of anxiety, depression and mixed common mental health problems. It compares the effectiveness of different types of brief psychological therapies within primary care across and between disorders compared to treatment as usual. In the absence of studies directly comparing different types of psychological therapy provided in primary care (with one exception [27]), such comparisons are needed to help inform decisions about treatment.

## Methods

### Search strategy

Studies were identified as part of a broader search of intervention studies in primary care mental health. The Medline, Embase and Psycinfo databases from inception to July 2008 were searched using a sensitive search strategy involving combinations of 'mental health' ('mental health' or psychol* or anx* or depress* or schizo* or dysthymi* or psychiatr* or emotion* or counsel*) and 'primary care' terms ('primary care' or 'primary health care' or 'family physician*' or 'practice nurs*' or 'general pract*' or GP*) in order to maximize identification of relevant interventions. Additional papers were identified from reference lists, from hand searching key journals and from contact with other primary care mental health researchers. All searches were limited to peer-reviewed published works in English.

### Inclusion criteria

For this review, we included published randomized controlled trials of brief psychological therapies for adult patients with anxiety, depression, unspecified common mental health problems or 'emotional distress' provided by someone other than the patient's general practitioner (GP) either in primary care or at home organized from primary care. 'Brief' was operationalized as more than two and less than 10 appointments, this number being a pragmatic choice on the grounds that it clusters around the six sessions commonly offered in primary care in the UK and is clearly fewer than the 12 - 24 sessions usually provided in secondary care efficacy trials. Studies of computerized or facilitated self-help [28-30], of psycho-educational groups [31,32], and of psychological therapy carried out as part of or referral on from case management within collaborative care [33], were excluded.

### Data extraction

Data from included studies were extracted into structured summary tables. Studies were classified according to type of psychological therapy and whether patients included had major depressive disorder (MDD), minor depression, mixed depression, anxiety or 'mixed anxiety and depression' (where participants with a range of diagnoses of anxiety, depression, unspecified common mental health problems or 'emotional distress' were included). Outcome data closest to 4 months from baseline were extracted where there was more than one follow-up period, as this was the most common follow-up interval used in our initial set of studies. Other information extracted included: details of the participants; study country; length of follow-up (weeks from baseline); number of treatment sessions; study design (individually randomized or cluster randomized); method of randomization and allocation concealment; use of intention-to-treat analysis; primary symptom outcome measure(s); data of publication; number of participants randomized and attrition from each group. The data were initially extracted by one reviewer, with the results being checked by a second reviewer who extracted the outcome data independently, but otherwise was not blinded to the findings of the first reviewer. Disagreements between reviewers were resolved by discussion.

The quality of each study was assessed by one reviewer who examined the adequacy of randomization and allocation concealment, and attrition using a modified version of the SIGN quality checklist for randomized controlled trials [34]. A second reviewer, blinded to the findings of the first reviewer, checked a sample of papers for reliability of the quality assessment.

### Meta-analysis

Comprehensive meta analysis (CMA) software, Version 2.2.040 [35] was used to calculate the standardized mean difference (*d*) and associated standard error for each study, computed from means and standard deviations (adjusted for baseline differences if reported) or from the sample size and *P*-value from an appropriate between-groups *t*- or *F*-test if no other data were reported. We used data from an intention-to-treat analysis, with last observation carried forward, rather than data from participants who completed the study, if both were reported. Where a study only reported data from dichotomous outcomes (remission or response to treatment), we assumed that participants who ceased to engage in the study - from whatever group - had an unfavourable outcome, then converted the log odds ratio into *d *using CMA. For the purposes of the review, negative values of *d *indicate that the outcome favoured the intervention. The metan command in Stata Version 9.2 [36] was used to produce forest plots and summary effects using a random-effects model.

We used *I*^2 ^and the *Q *test of heterogeneity [37,38] to examine among-study variation in the meta-analysis. Significant variation was confirmed by visual inspection of the forest plots. *I*^2 ^describes the proportion of total variation in study effect sizes that is due to heterogeneity as opposed to sampling error, with 25%, 50% and 75% indicating low, moderate and high heterogeneity [38].

We conducted planned sub-group analyses based on both type of psychological therapy and diagnosis. Sensitivity analyses were used to examine how robust these findings were to assumptions made when calculating effect size.

To check for publication bias, CMA was used to generate funnel plots and Egger's regression asymmetry test [39]. Where asymmetry was detected, we assessed the potential impact of the publication bias using the Duval and Tweedie nonparametric 'trim and fill' method [40]. This method recalculates the effect size given the presence of publication bias.

### Meta-regression

We used the metareg command in Stata, to conduct random-effects meta-regression analyses with restricted maximum likelihood estimation and the improved variance estimator of Knapp and Hartung [41]. Where data allowed, univariate models were used to examine whether there were differences between psychological therapies and between diagnostic categories in the magnitude of the treatment effect. In addition, where possible, we used multivariate models to control for the following study characteristics if they were shown to be potential moderators in univariate models: country; year of publication; number of sessions; total number of participants randomized; type of data (continuous versus dichotomous); allocation concealment; use of intention-to-treat analysis; and attrition. In our analyses, the regression coefficients are the estimated change in *d *per unit change in each covariate.

## Results

**Figure 1:**
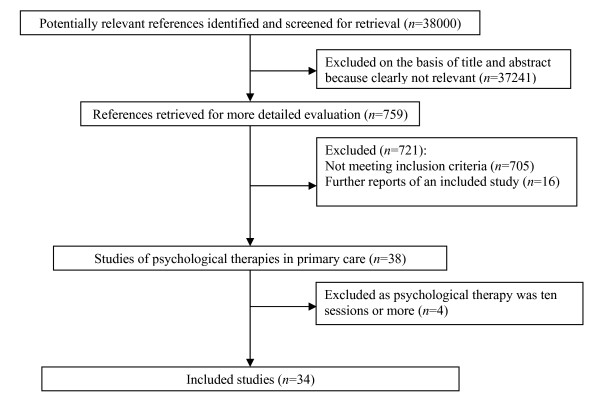
**Results of literature searches and selection of randomized controlled trials for inclusion in the meta-analyses**.

The flowchart outlining the search process is shown in Figure 1. It should be noted that this represents the entirety of the search, of which only a proportion related to psychological therapies in primary care. Thirty-four studies met our inclusion criteria. There were four studies excluded on basis of the psychological therapy being 10 sessions or more [42-45].

Details of the included studies are given in Table 1. Twelve were of cognitive behaviour therapy (CBT) [46-57], seven of counselling [58-64], one of interpersonal psychotherapy [65], one of psychodynamic psychotherapy [66] and 12 of problem solving therapy (PST) [67-78]. One study included both a CBT and a counselling intervention arm compared to a usual GP treatment control [27] and so in the meta-analysis the control group was halved to avoid double counting.

**Table 1:** Details of included studies

Study name	Study characteristics								Quality assessment				
	**Tx**	**Country**	**Diag**	**Outcome**	**No. sessions**	**Type of data**	**FU (weeks)**	**Total N**	**Rand**.	**AC**	**ITT**	**% attrition (Intv)**	**% attrition (Ctl)**

Barrett 2001 [67]	PST	USA	Dep - minor or dys	HDRS < 7	6	D	11	161	WC	WC	Y	18	16
Boot 1994 [58]	COU	UK	Mixed Anx and Dep	GHQ	6	C	6	192	AA	U	N	46	40
Brodaty 1983 [66]	PP	AUS	Mixed Anx and Dep	GHQ	8	C	8	78	AA	U	N	65	26
Catalan 1991 [68]	PST	UK	Mixed Anx and Dep	PSE	4	C	11	47	AA	U	N	0	0
Dowrick 2000 [69]	PST	EU	Dep - MDD or dys	BDI	6	C	26	317	AA	WC	Y	23	26
Earll 1982 [46]	CBT	UK	Mixed Anx and Dep	DSSI/sAD *	7.7	D	32	50	AA	U	N	16**	
Friedli 1997 [59]	COU	UK	Mixed Anx and Dep	BDI	9	C	12	136	AA	AA	Y	16	23
Harvey 1998 [60]	COU	UK	Mixed Anx and Dep	HADS	6	C	16	162	AA	U	Y	31	25
Hemmings 1997 [61]	COU	UK	Mixed Anx and Dep	MHSI	5.7	C	16	188	AA	U	N	16	23
Holden 1989 [62]	COU	UK	Dep - MDD	RDC	8	D	12	50	AA	U	N	0	0
Kendrick 2005 [70]	PST	UK	Mixed Anx and Dep	CIS-R	6	C	26	168	WC	WC	Y	20 †	31†
Lang 2006 [71]	PST	USA	Mixed Anx and Dep	BSI-D	4	C	12	62	AA	U	Y	34	17
Lindsay 1987 [47]	CBT	UK	Anx - GAD	GHQ-28	8	C	4	20	AA	U	N	0	0
Liu 2007 [72]	PST	TA	Mixed Anx and Dep	CIS-R	2.27	C	16	169	WC	AA	Y	25	22
Lynch 1997 [73]	PST	USA	Dep - minor	HDRS	6	C	7	29	AA	U	N	27	7
Lynch 2004 [74]	PST	USA	Dep - minor	HDRS	6	C	6	36	AA	U	N	50	28
Marks 1985 [48]	CBT	UK	Anx - phobic	PS	6	C	26	92	AA	U	N	37	20
Mynors-Wallis 1995 [75]	PST	UK	Dep - MDD	HDRS	6	C	12	55	AA	U	Y	0	0
Mynors-Wallis 1997 [76]	PST	UK	Mixed Anx and Dep	CIS	4.5	C	26	70	AA	AA	N	20	13
Power 1989 [49]	CBT	UK	Anx - GAD	HAM-A	4	C	6	21	AA	U	Y	0	0
Power 1990 [51]	CBT	UK	Anx - GAD	CGI = 1	7	D	10	79	AA	U	N	0	0
Power 2000 [50]	CBT	UK	Anx - panic	HAM-A	6	C	12	72	AA	U	N	16	17
Prendegast 2001 [52]	CBT	AUS	Dep - mixed	EPDS < 10	6	D	26	37	AA	U	N	0	0
Robson 1984 [53]	CBT	UK	Mixed Anx and Dep	PS	3.7	C	14	429	AA	U	N	0 ††	0 ††
Schreuders 2007 [77]	PST	NL	Mixed Anx and Dep	HADS	6	C	12	175	AA	WC	N	30	22
Scott 1992 [54]	CBT	UK	Dep - MDD	HDRS	9.8	C	16	60	AA	AA	Y	3	3
Scott 1997 [55]	CBT	UK	Dep - MDD	HDRS	6	C	7	48	AA	U	N	25	33
Sharp 1996 [56]	CBT	UK	Anx - panic	HAM-A ‡	7	D	13	154	AA	U	Y	21^§^	24§
Sharp 2004 [57]	CBT	UK	Anx - panic	HAM-A	8	C	12	59	AA	U	Y	16	14
Simpson 2003 [63]	COU	UK	Dep - mixed	BDI	5	C	26	145	AA	U	Y	11	10
Van Schaik 2006 [65]	IPT	NL	Dep - MDD	MADRS	8	C	26	143	WC	WC	Y	16	16
Ward 2000 [27]	CBT	UK	Dep - mixed	BDI	5	C	16	197	AA	U	Y	11	8
	COU				6.4							8	
Wickberg 1996 [64]	COU	SW	Dep - mixed	MADRS ¶	6	D	7	45	AA	U	N	0	0
Williams 2000 [78]	PST	USA	Dep - minor or dys	HDRS < 7	6	D	11	278	WC	AA	Y	18^§^	15^§^

* Patients were categorized as not 'personally ill' by study authors.

** Number randomized to each group not reported by study authors.

^† ^We used missing data that were imputed from a regression analysis that took account of the baseline information for each participant with missing data rather than from last observation carried forward because of differential attrition between study groups.

^†† ^Attrition not reported by study authors; therefore we assumed there was no missing data.

^‡ ^Study authors defined response as a clinically significant change.

^§ ^For the purposes of the meta-analysis, participants with missing data were assumed to have had a poor outcome.

^¶ ^Study authors defined response as a substantial improvement.

AA, adequately addressed; AC, allocation concealment; AUS, Australia; Anx, anxiety; BDI, Beck Depression Inventory; BSI-D, Brief Symptom Inventory - Depression scale; C, continuous; CBT, cognitive behavioural therapy; CES-D, Center for Epidemiologic Studies Depression Scale; CGI, Clinical Global Impression; CIS-R, Clinical Interview Schedule - Revised; COU, Counselling; Ctl, control; D, dichotomous; Dep, depression; Dep - mixed, mixed depressive diagnoses including minor depression; Diag, diagnosis; DSSI/sAD, Delusions-Symptoms-States Inventory/states of Anxiety and Depression; dys, dysthymia; EPDS, Edinburgh Postnatal Depression Scale; EU, European countries; GHQ, General Health Questionnaire; HADS, Hospital Anxiety and Depression Scale; HAM-A, Hamilton Anxiety Rating Scale; HDRS, Hamilton Depression Rating Scale; Intv, intervention; IPT, Interpersonal psychotherapy; ITT, intention-to-treat analysis; MADRS, Montgomery-Åsberg Depression Rating Scale; MDD, major depressive disorder; MHSI, Mental Health Symptom index;; Mixed Anx and Dep, mixed anxiety and depression diagnoses; N, number randomized; NL, Netherlands; PP, Psychodynamic psychotherapy; PS, psychiatric symptoms; PSE, Present State Examination; PST, problem solving therapy; Rand., randomization; RDC, Research Diagnostic Criteria; SW, Sweden; TA, Taiwan; Tx, treatment; U, unclear; WC, well covered; Y, yes.

Of the 34 studies, 14 were of patients with depression (six MDD, four minor depression, four mixed depression), seven were of anxiety disorders (three generalized anxiety disorder, three panic disorder, one mixed phobic disorders) and 13 studies were of patients with 'mixed anxiety and depression' (including patients with a range of diagnoses of anxiety, depression and unspecified common mental health problems).

Twenty-two of the 34 studies were carried out in UK primary care, five in the USA, two in the Netherlands, two in Australia, one in Sweden, one in Taiwan and one was a multi-site study in five European countries. All studies were randomized by the individual participant. Seven studies were published in the 1980s, 13 in the 1990s and 14 in the present decade. In 14 studies the psychological therapy was conducted in the patients' usual general practice or primary care clinic location, in two studies over the telephone, in three studies at home, in four studies both at home and in other primary care settings and in 11 studies the psychological therapy was described as being carried out 'in a primary care setting', 'in a local health centre' or 'in primary care' without this being further specified. The control condition for all studies was usual GP care, supplemented in a few studies [49-51,56,67,75,78] by the patient receiving some additional control intervention, for example placebo medication or a self-help booklet.

There was similar median treatment intensity of six to seven contacts with the patient in CBT, counselling and PST, with the single studies of interpersonal psychotherapy and psychodynamic psychotherapy each involving eight sessions of treatment. In terms of the length of follow up, there was some variation between psychological therapies (median 14 weeks for counselling, 13 weeks for CBT, and 12 weeks for PST) and also between the CBT for anxiety (12 weeks) and the CBT for depression studies (16 weeks).

Table 1 also gives details of the quality assessment of each study. The method of randomization was well covered (15% of studies) or adequately addressed (85%). Allocation concealment was unclear (71%) in most studies. Sixteen (47%) studies were analysed by intention-to-treat (ITT), with the remainder either not using ITT analysis or this being unclear in the reported paper. Twenty-one (62%) studies reported less than 20% attrition across both groups with eight studies (24%) reporting no attrition. In two studies [62,70] there was 50% or more attrition from either group.

### Effect size of psychological therapies

**Figure 2:**
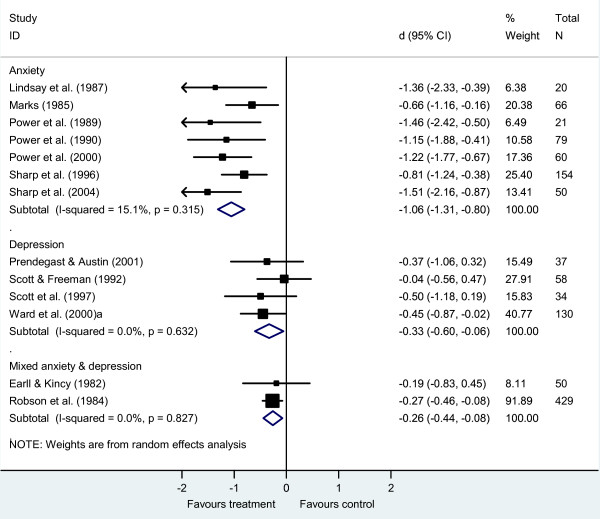
**Brief cognitive behaviour therapy versus usual general practitioner care, sub-grouped by diagnosis**.

The meta-analysis showed small effects favouring brief CBT over usual GP care for both depression [*d *-0.33, 95% confidence interval (CI) -0.60 to -0.06, *k *= 4, *n *= 450) and mixed anxiety and depression (*d *-0.26, 95% CI -0.44 to -0.08, *k *= 2, *n *= 479) and a larger effect for brief CBT for anxiety disorders (anxiety *d *-1.06, 95% CI -1.31 to -0.80, *k *= 7, *n *= 450; Figure 2). In each meta-analysis, heterogeneity between studies was low (CBT for depression *I*^2 ^= 0%, *Q *= 1.72, *P = 0*.63; CBT for mixed anxiety and depression *I*^2 ^= 0%, *Q *= 0.05, *P =*.83; CBT for anxiety *I*^2 ^= 15%, *Q *= 7.07, *P = 0*.32).

**Figure 3:**
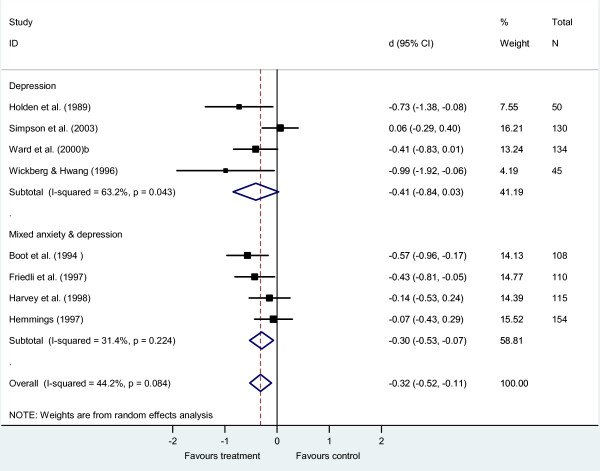
**Brief counselling *versus *usual general practitioner care, sub-grouped by diagnosis**.

The meta-analysis suggested counselling was effective for mixed anxiety and depression (*d *-0.30, 95% CI -0.53 to -0.07, *k *= 4, *n *= 487), while the effect size for counselling for depression, although similar in size, fell short of statistical significance (*d *-0.41, 95% CI -0.84 to 0.03, *k *= 4, *n *= 359; Figure 3). Heterogeneity between studies was moderate to high for the studies of depression (*I*^2 ^= 63%, *Q *= 8.15, *P = 0*.04) and low for mixed anxiety and depression (*I*^2 ^= 31%, *Q *= 4.37, *P = 0*.22). There were no studies of counselling for anxiety disorders. Pooling across the studies of depression and mixed anxiety and depression, heterogeneity was intermediate (*I*^2 ^= 44%, *Q *= 12.55, *P = 0*.04), with a small effect favouring counselling over usual GP care (*d *-0.32, 95% CI -0.52 to -0.11, *k *= 8, *n *= 846).

**Figure 4:**
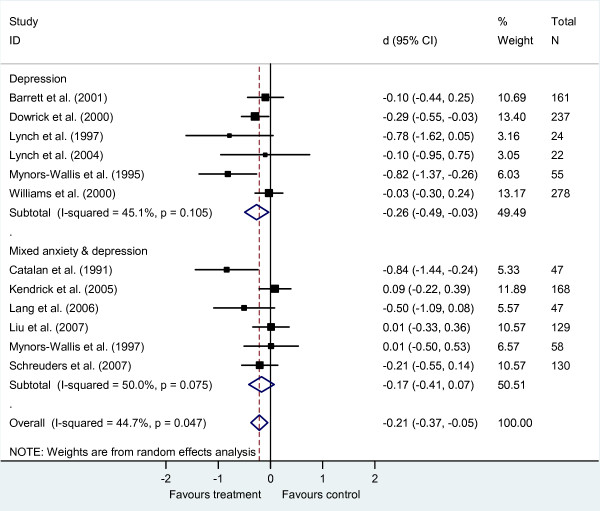
**Brief problem solving therapy versus usual general practitioner care, sub-grouped by diagnosis**.

The effect size was smaller for PST for both depression (*d *-0.26, 95% CI -0.49 to -0.03, *k *= 5, *n *= 777) and mixed anxiety and depression (*d *-0.17, 95% CI -0.41 to -0.07, *k *= 6, *n *= 579; Figure 4). Heterogeneity between studies approached moderate for the studies of depression (*I*^2 ^= 45%, *Q *= 9.11, *P = 0*.11) and was moderate for mixed anxiety and depression (*I*^2 ^= 50%, *Q *= 10.01, *P = 0*.08). There were no studies of PST for anxiety disorders. Pooling across all studies produced a small effect favouring PST over usual GP care (*d *-0.21, 95% CI -0.37 to -0.05, *k *= 12, *n *= 1356) with heterogeneity approaching a moderate level (*I*^2 ^= 45%, *Q *= 19.88, p = 0.05).

The evidence for interpersonal psychotherapy (*d *-0.11, -0.47 to 0.24, *k *= 1, *n *= 120) and psychodynamic psychotherapy (*d -*0.01, 95% CI = -0.64 to 0.63, *k *= 1, *n *= 38) was inconclusive, although only one small study was included for each of these in this review.

For the studies of depression and mixed anxiety and depression, a series of random-effects meta-regressions comparing types of psychological therapy, controlling for diagnosis, indicated there was no difference between CBT (*k *= 6) and counselling (*k *= 8; regression coefficient -0.02, *P =*.91, adjusted *R*^2 ^= 0%, *n *= 1584) or between CBT (*k *= 6) and PST (*k *= 12) (regression coefficient 0.10, *P = 0*.45, adjusted *R*^2 ^= 0%, *n *= 2094) or between counselling (*k *= 8) and PST (*k *= 12) (regression coefficient 0.10, *P = 0*.46, adjusted *R*^2 ^= 0%, *n *= 2202).

### Impact of diagnosis

We conducted two sets of meta-regression analyses to explore the effect of diagnosis on the magnitude of the treatment effect. First, we looked at whether there was a difference between studies of anxiety, depression and mixed disorders in the 13 studies of CBT (Figure 2; Table 2). This analysis was limited to studies of CBT as all studies of anxiety disorders were of CBT. The results indicate that there was a statistically significant difference favouring the studies of anxiety (*k *= 7) over depression (*k *= 4; regression coefficient 0.72, *P *= 0.005, adjusted *R*^2 ^= 91%, *n *= 709). There was also a significant difference favouring the studies of anxiety (*k *= 7) over mixed anxiety and depression (*k *= 2; regression coefficient 0.79, *P *= 0.003, adjusted *R*^2 ^= 94%, *n *= 929). There was no difference between the studies of depression (*k *= 4) and mixed anxiety and depression (*k *= 2; regression coefficient 0.07, *P *= 0.70, adjusted *R*^2 ^= 0%, *n *= 738). In accordance with our planned analytic strategy, as no study characteristic other than diagnosis predicted effect size of CBT studies in the univariate models (Table 2), we did not use a multivariate meta-regression model.

**Table 2:** Results of univariate meta-regressions for the 13 studies of cognitive behaviour therapy

Variable	Category 1 (*k*; *N*)	Category 2 (*k*; *N*)	Regression coefficient	Standard error	95% CI	P*	***I***^**2**^	**Adj *R***^**2**^
**Dichotomous**								
Diagnosis (model a)	Anxiety = 0 (7; 497)	Depression = 1 (4; 275)	.72	.20	.28-1.16	.005	0%	91%
Diagnosis (model b)	Anxiety = 0 (7; 497)	Mixed anxiety & depression = 1 (2, 479)	.80	.18	.36-1.23	.003	2%	94%
Diagnosis (model c)	Depression = 0 (4; 275)	Mixed anxiety & depression = 1 (2; 479)	.07	.17	-.39-.53	.70	0%	0%
Country	UK = 0 (12; 1214)	Other = 1 (1; 37)	.36	.56	-.87-1.58	1.00	71%	0%
Type of data	Continuous = 0 (9; 931)	Dichotomous = 1 (4; 320)	-.11	.31	-.79-.58	1.00	70%	0%
**Continuous**	**Range (*k*; *N*)**							
Number of sessions	3.7-9.8 (13; 1251)		-.03	.08	-.21-.15	1.00	65%	0%
Follow up (weeks)	4-31.6 (13; 1251)		.03	.02	-.003-.07	.46	67%	22%
Year of publication	1982-2004 (13; 1251)		-.02	.02	-.06-.02	.89	59%	16%
Number randomized	20-429 (13; 1251)		.001	.001	-.001-.004	.89	59%	7%
Attrition (intervention group)	0-37% (13; 1251)		-.01	.01	-.03-.02	1.00	67%	0%
Attrition (control group)	0-33% (13; 1251)		-.01	.01	-.04-.03	1.00	67%	0%
Study quality	1-4 (13, 1251)		.16	.22	-.31-.64	1.00	71%	0%

*P-values are adjusted for multiple testing, calculated using the Higgins and Thompson Monte Carlo permutation test (10,000 permutations), except for the diagnostic variables which are unadjusted.

Adj *R*^2 ^= adjusted *R*^2 ^(proportion of between-study variance explained by the covariate); *k *= number of studies; *N *= total sample size.

Translating the effect sizes for CBT for anxiety and depression into Hamilton rating scale equivalents to give an indicator of clinical significance [using all studies in the database to estimate the Hamilton standard deviations], the effect size for CBT for depression was equivalent to a 2.3 point difference between groups on the Hamilton Rating Scale for Depression [79] while the effect size for CBT for anxiety was equivalent to 7.2 points on the Hamilton Rating Scale for Anxiety [80].

In the second set of meta-regressions, we examined whether the type of depression diagnosis had an impact on the treatment effect across types of psychological therapy. The results indicate that there was no significant difference between MDD (*k *= 6) and minor depression/mixed depression (*k *= 9; regression coefficient 0.12, *P *= 0.43, adjusted *R*^2 ^= 2%, *n *= 1515), or between minor depression (*k *= 4) and MDD/mixed depression (*k *= 11; regression coefficient -0.20, *P *= 0.22, adjusted *R*^2 ^= 25%, *n *= 1515). No other study characteristic predicted effect size of the depression studies, so no multivariate meta-regression was used.

### Publication bias

Funnel plots of the CBT studies, showed evidence of asymmetry in the studies of CBT for anxiety (Egger's test, one-tailed *P *= 0.04), but not CBT for depression/mixed anxiety and depression (*P *= 0.38). There was also evidence of asymmetry in the studies of counselling (one-tailed *P *= 0.03) and PST (one-tailed *P *= 0.03). The Duval and Tweedie 'trim and fill' method suggested that, for CBT for anxiety, three studies were potentially missing and, if imputed, the overall summary effect would drop to *d *-0.91 (95% CI -1.18 to -0.63). For counselling, imputing three missing studies reduced the effect size to *d *-0.19 (95% CI -0.41 to -0.04). For PST, imputing two missing studies reduced the effect size to *d *-0.14 (95% CI -0.32 to -0.05).

## Discussion

### Summary of main findings

The majority of studies of brief psychological therapies for anxiety and depression in primary care included in this review were of CBT, counselling and PST, with a single study each of interpersonal psychotherapy and of psychodynamic psychotherapy. The meta-analysis suggests that brief CBT, counselling and PST were all effective. No significant difference was found between CBT, counselling and PST on meta-regression, when controlling for diagnosis,

Brief CBT for anxiety (mostly generalized anxiety disorder and panic disorder) had a greater impact on clinical outcomes than brief CBT for depression or of mixed groups of patients with common mental health problems, for which the outcomes were similar to studies of counselling and of low clinical significance. There were no studies of counselling or PST for anxiety disorders alone, so it is not possible to establish from this review whether this is a specific differential effect of CBT or whether other brief psychological therapies might also have greater effects on anxiety than depression.

### Comparison with existing literature

Reviews of CBT have generally found larger effect sizes compared to control for CBT as a treatment for anxiety disorders, with smaller effects obtained for CBT as a treatment for depression [12]. This is similar to the differential effect for brief CBT found in the present review. A recent review of internet-based CBT of anxiety and depression, also found differential effects between studies of internet-based CBT on depression and studies of internet-based CBT on anxiety [81], with remarkably similar effect sizes (*d *0.27 depression, *d *0.93 anxiety) to those in the present review (*d *0.33 depression, *d *1.06 anxiety).

The summary effects obtained were generally lower than reviews of secondary care based treatments, involving a longer duration of psychological therapies [10,12,82]. These differences in effect size could be due to a number of factors: length of treatment, type of included patients, training of therapists or location of treatment. In terms of type of included patients, participants in these primary care based studies may have had less severe conditions than those in secondary care based studies which would correspondingly limit the potential effect sizes. Brief CBT for anxiety disorders was the exception, with effect sizes in the present review comparable to those obtained in reviews of longer secondary care based treatments [12,13]. This may not be unique to primary care. In secondary care, a direct comparison of brief and standard length CBT for panic disorder found equivalent effectiveness [83].

### Strengths and limitations of the study

The strengths of the review are the inclusion of studies of representative populations of primary care patients, including with mixed anxiety and depression, and the use of meta-regression to compare effectiveness between different types of problems and different types of psychological therapy.

Limitations are the restriction to published studies and to English language publications. Other relevant studies may have been missed, particularly negative studies leading to an overestimation of the effects of brief psychological therapies. We did find evidence of possible publication bias and that accounting for this would have reduced effect sizes, although not changed the key conclusions of the review. Type of outcome measures, number of treatment sessions, follow-up intervals, country, number of participants randomized and aspects of study quality varied between studies, increasing heterogeneity and, hence, decreasing the likelihood of finding differences between types of psychological therapy and different diagnoses. Meta-regression ideally requires large numbers of studies and the sample size of studies in the review may have been too small to show other than relatively large effect size differences between types of psychological therapy.

The majority of studies in the meta-analysis used questionnaires and rating scales as outcome measures. Although this is standard in measurement of depression and anxiety outcomes, responses to such measures can vary between gender, language, culture and setting and are only a proxy for diagnosis. When analysed as continuous measures, there are potential problems caused by lack of interval-scaling, which may result in a sigmoidal, rather than linear, relationship between the score and the underlying trait [84]. Dependence on such measures in the meta-analysis is likely to have increased measurement error and heterogeneity [85]. They may also have led to systematic biases in the meta-regression where groups being compared (for example, patients with MDD versus minor and mixed depression) were using different measures or had baseline differences on the same measure.

A further limitation is the likely variation in locations included as 'primary care'. Reviews of psychological therapies in primary care vary in definitions as to what is included as primary care, with some reviews including studies if patients are recruited in or referred from primary care irrespective of where patients are treated. We set out to include studies where patients were treated either in a primary care setting or at home organized from primary care, but many study reports lacked details of where patients were seen other than 'in a primary care setting'. The significance of treatment in primary care is considered to be familiarity and accessibility of location and ease of liaison between GP and treating psychological therapist but this will quite probably have varied widely, given that studies varied from only one or two patients treated per participating general practice [55,56,75], to a few hundred [53]. Better reporting of location of treatment and nature of liaison with patients' GPs should be encouraged in studies of treatment in primary care.

## Conclusions

This review confirms the effectiveness of brief CBT, counselling and PST for routine delivery in primary care but with the caution that effect sizes are low when compared to patients receiving these treatments over a longer duration, so for many patients brief treatments may not be sufficient. The exception is brief CBT for anxiety disorders, which was comparable in effectiveness to longer treatments. While this suggests that brief CBT is particularly effective with anxiety disorders and there is evidence that training in CBT may enhance effectiveness of treatment of anxiety disorders by counsellors [86], the lack of randomized studies of brief psychological therapies other than CBT for patients suffering from anxiety disorders means that it is not possible to definitively determine whether brief CBT is more effective than other brief psychological treatments for anxiety disorders within primary care.

## Abbreviations

CBT: cognitive behaviour therapy; CMA: comprehensive meta analysis; GP: general practitioner; ITT: intention-to-treat; MDD: major depressive disorders; PST: problem solving therapy.

## Competing interests

The authors declare that they have no competing interests.

## Authors' contributions

JC, MB, PW and CW conceived and designed the study. JC contributed to the literature searches. CW, JC and LU extracted the data. CW analysed the data. JC wrote the initial draft of the manuscript. MB, PW and CW contributed to the revision of the manuscript. All authors read and approved the final manuscript.

## Pre-publication history

The pre-publication history for this paper can be accessed here:

http://www.biomedcentral.com/1741-7015/8/38/prepub
